# Atherogenic dyslipidemia and risk of silent coronary artery disease in asymptomatic patients with type 2 diabetes: a cross-sectional study

**DOI:** 10.1186/s12933-016-0415-4

**Published:** 2016-07-22

**Authors:** Paul Valensi, Antoine Avignon, Ariane Sultan, Bernard Chanu, Minh Tuan Nguyen, Emmanuel Cosson

**Affiliations:** Department of Endocrinology-Diabetology-Nutrition, CRNH-IdF, CINFO, AP-HP, Jean Verdier Hospital, Université Paris 13, Sorbonne Paris Cité, Avenue du 14 Juillet, 93143 Bondy Cedex, France; Unité de Recherche Epidémiologique Nutritionnelle, UMR U1153 INSERM/U11125 INRA/CNAM/Université Paris 13, 93000 Bobigny, France; Department of Endocrinology-Diabetology-Nutrition, CHRU Montpellier, 34295 Montpellier Cedex 5, France; PhyMedExp, INSERM U1046, CNRS UMR 9214, University of Montpellier, 34295 Montpellier Cedex 5, France

**Keywords:** Silent myocardial ischemia, Diabetes, Residual cardiovascular risk, Atherogenic dyslipidemia, Triglycerides, HDL-cholesterol

## Abstract

**Background:**

To investigate whether atherogenic dyslipidemia, a dyslipidemic profile combining elevated triglycerides and low high-density lipoprotein (HDL) cholesterol, is predictive of risk of silent myocardial ischemia (SMI) or angiographic coronary artery disease (CAD) in asymptomatic patients with type 2 diabetes.

**Methods:**

Cohort study in 1080 asymptomatic patients with type 2 diabetes with a normal resting electrocardiogram, at least one additional cardiovascular risk factor and low density lipoprotein (LDL) cholesterol <3.35 mmol/L. Patients initially underwent screening for SMI by stress myocardial scintigraphy. Patients with SMI underwent coronary angiography.

**Results:**

Overall, 60 (5.5 %) patients had atherogenic dyslipidemia (triglycerides ≥2.26 mmol/L and HDL cholesterol ≤0.88 mmol/L). In multivariate analyses taking into account the parameters associated in univariate analyses with SMI and then CAD, atherogenic dyslipidemia was associated with SMI (odds ratio 1.8[1.0–3.3]), as were male gender (OR 2.1[1.5–2.9]), BMI (OR 0.97[0.94–0.997]), retinopathy (OR 1.4[1.1–1.9]), peripheral occlusive arterial disease (POAD: OR 2.5[1.6–3.8]) and mean blood pressure (OR 1.01[1.00–1.03]); atherogenic dyslipidemia was associated with CAD (OR 4.0[1.7–9.2]), as were male gender (OR 3.0[1.6–5.6]), BMI (OR 0.94[0.90–0.995]), retinopathy (OR 1.7[1.0–2.9], POAD (OR 4.0[2.1–7.4]) and mean blood pressure (OR 1.03[1.01–1.05]). In the subgroup of 584 patients with LDL cholesterol <2.6 mmol/L, atherogenic dyslipidemia was also associated with CAD (OR 3.6[1.5–9.0]).

**Conclusions:**

Atherogenic dyslipidemia was associated with an increased risk of SMI and silent CAD in patients with type 2 diabetes and LDL cholesterol levels <3.35 mmol/L. Specific management of atherogenic dyslipidemia might help reducing the high residual burden of cardiovascular disease.

## Background

Coronary artery disease (CAD) is the main cause of death and morbidity in patients with type 2 diabetes. However, symptomatic events represent only a part of this burden, as asymptomatic disease is probably at least as common with a high prevalence of silent myocardial infarction [1] and silent myocardial ischemia (SMI) [2], especially in those with additional cardiovascular risk factors [3–5]. Between one- and two-thirds of the patients with SMI also have angiographic evidence of coronary artery disease (CAD) [3]. SMI is strongly predictive of cardiovascular events and poorer outcomes [3, 6] above and beyond routine risk predictors [7]. There is a strong need for understanding the risk factors for SMI, which in turn might improve early diagnosis and treatment.

Intensive multifactorial intervention targeting blood pressure, glycemia and low-density lipoprotein (LDL) cholesterol remains the cornerstone of type 2 diabetes management, with a substantial 50 % reduction in the risk of cardiovascular events [8]. However, there is a need to identify patients with residual risk and to target new cardiovascular risk factors. Lipid abnormalities usually persist in statin-treated CAD patients with or without diabetes [9]. High triglycerides levels are associated with high cardiovascular risk [10], the presence of CAD [11] and a higher mortality [12, 13]. Adjustment for high-density lipoprotein (HDL) cholesterol levels tends to attenuate this association [13]. Alterations in lipid transfers to HDL have been reported in patients with type 2 diabetes and CAD [14]. Further, low HDL cholesterol levels are associated with CAD in patients submitted to coronary angiography [11] as are non-HDL-to-HDL-cholesterol ratio [15] and triglycerides-to-HDL-cholesterol ratio [16] with coronary heart disease in type 2 diabetes.

Higher triglyceride and lower HDL cholesterol levels, considered separately only, were reported in little series of subjects with type 2 diabetes and SMI or silent CAD [17–19]. The combination of elevated triglyceride level and low HDL cholesterol level, commonly named atherogenic dyslipidemia, may therefore be a contributing factor to SMI, in particular in patients with LDL cholesterol in the target range [20–23]. Furthermore, controlling atherogenic dyslipidemia in these patients might improve their cardiovascular prognosis [20, 24–26]. Consequently, we made the hypothesis that atherogenic dyslipidemia was associated with SMI and CAD in asymptomatic patients with type 2 diabetes. We also explored the association between non HDL cholesterol and silent myocardial ischemic status.

## Methods

### Patients

The patients were consecutively recruited between 1991 and 2011 in the diabetes clinics of Jean Verdier Hospital, Bondy and Lapeyronie Hospital, Montpellier, France. The data are retrospective and observational, with no need for either approval by an ethics committee/institutional review board or patients’ written informed consent. The patients’ records/information are anonymous. Type 2 diabetic patients were included in the cohorts if they were asymptomatic, did not have heart failure, and had a normal 12-lead resting electrocardiogram (ECG), and at least one additional cardiovascular risk factor among dyslipidemia (total cholesterol >6.5 mmol/L and/or LDL-cholesterol >4.1 mmol/L and/or HDL-cholesterol <0.9 mmol/L and/or triglycerides >2.3 mmol/L and/or lipid lowering medication), hypertension (blood pressure ≥140/90 mmHg or anti-hypertensive treatment), smoking, nephropathy (urinary albumin excretion rate >30 mg/day on at least two measurements and/or estimated glomerular filtration rate <60 mL/min), family history of premature CAD (before the age of 60 in first-degree relatives), peripheral occlusive arterial disease (POAD: stenosis measured ≥50 % by ultrasound examination). Patients with a history of valvular heart disease, congenital heart disease or cardiomyopathy, or symptomatic CAD were excluded. In this study, we selected the patients with LDL cholesterol levels <3.35 mmol/L, which was the target range before 2000. We also selected a subgroup with LDL cholesterol values <2.6 mmol/L, with or without lipid-modifying treatment.

### Methods

At baseline, clinical examination was performed and the presence of diabetic complications (retinopathy, nephropathy, peripheral neuropathy, POAD) was assessed. Patients initially underwent screening for type 1 SMI by ^201^thallium myocardial scintigraphy after an ECG stress test, a pharmacological stress test (dipyridamole injection), or both, as previously reported [3, 7, 27]. ECG stress test was performed when the patient was able to exercise on a bicycle ergometer and was expected to have an interpretable exercise ECG. If the patient was unable to exercise or when the ECG stress test result was indeterminate, a pharmacological stress test was performed. SMI was defined by evidence of an abnormal ECG stress test and/or abnormal myocardial scintigraphy imaging (i.e., defects in at least 3 out of 17 segmental regions). The patients with SMI were subsequently screened for angiographic coronary artery disease. CAD was defined as ≥70 % narrowing of the luminal diameter in the left anterior descending artery, the circumflex artery, a well-developed marginal vessel or the right coronary artery or ≥50 % narrowing of the left main coronary artery diameter. The patients with SMI but who did not undergo a coronary angiography were not considered in analyzing the factors associated with CAD.

Blood samples were taken for measurement of HbA1c (Dimension^®^ technology, Siemens Healthcare Diagnosis Inc., Newark, USA), serum total and HDL cholesterol and triglycerides (enzymatic colorimetry, Hitachi 912, Roche Diagnostics, Meylan, France), serum creatinine (colorimetry, Kone Optima, Thermolab System, Paris La Défense, France). Urine samples were collected for measurement of 24-h proteinuria and 24-h urinary albumin excretion rate (laser immunonephelometry, BN100, Dade-Behring, Paris, France). LDL cholesterol was calculated according to the Friedewald formula and glomerular filtration rate was estimated using the Modification of Diet in Renal Disease (MDRD) Study equation. Atherogenic dyslipidemia was defined in accordance with the criteria used in the ACCORD Lipid study [20] as the combination of low plasma levels of HDL cholesterol (≤0.88 mmol/L [34 mg/dL]) and elevated triglycerides (≥2.26 mmol/L) [204 mg/dL]. Non HDL cholesterol was also calculated. Apoproteins A1 and B were measured in the population of Bondy center (immunoturbidimetry, COBAS 6000, Roche Diagnostics, Meylan, France).

### Statistical analyses

We hypothesized that the presence of atherogenic dyslipidemia would be associated with an increased risk for SMI or silent CAD. This was first investigated for the patients with LDL cholesterol <3.35 mmol/L and then in the subgroup of patients with LDL cholesterol below the target value (<2.6 mmol/L). No data replacement procedure was used for missing data. Continuous variables were expressed as mean ± SD and compared by one-way ANOVA or the Mann–Whitney’s *U* test as adequate. The significance of differences in proportions was tested with the χ^2^ test. Logistic regression was used for multivariate analyses based on models including the factors that were associated with silent coronary status with a p value ≤0.10 in univariate analyses. Odds ratios with 95 % confidence intervals (95 % CI) for the risk of SMI or CAD were reported. We calculated the Hosmer–Lemeshow χ^2^ statistic (HL χ^2^) to test the goodness of fit between expected and observed probabilities of an event in the different models. Statistical analyses were carried out using SPSS software (SPSS, Chicago, IL). The level of significance for all tests was p < 0.05.

## Results

### Patient characteristics

A total of 1623 patients with type 2 diabetes were screened for SMI. We selected 1080 patients who had LDL cholesterol levels <3.35 mmol/L. Their baseline characteristics are summarized in Table 1. Overall, patients had long-standing diabetes (mean 13.8 years), with a high a priori cardiovascular risk: hypertension (76.3 %), nephropathy (57.3 %), smoking habits (22.8 %), familial history of premature CAD (13.0 %) and POAD (11.3 %). LDL cholesterol was 2.4 ± 0.6 mmol/L. The 543 patients who were not included had significantly higher HbA1c (8.9 ± 2.5 %; 74 ± 8 mmol/mol), more dyslipidemia (72.1 %) and less nephropathy (30.8 %) and statin treatment (25.0 %), with no difference for other parameters (Table 2). Finally, 584 patients (5.4 %) had baseline LDL cholesterol levels below the target value (<2.6 mmol/L).

**Table 1:** Patients’ characteristics according to the presence or absence of atherogenic dyslipidemia

	Total n = 1080	No atherogenic dyslipidemia n = 1020	Atherogenic dyslipidemia n = 60	p
Clinical characteristics				
Age, years	61.6 ± 9.0	61.7 ± 9.1	59.7 ± 8.2	0.09
Gender, male/female	614/466	577/443	37/23	0.44
Body mass index, kg/m^2^	30.2 ± 5.9	30.2 ± 6.0	31.1 ± 4.9	0.23
Waist circumference, cm	104 ± 13	104 ± 13	107 ± 10	0.37
Diabetes				
Diabetes duration, years	13.8 ± 8.5	13.9 ± 8.6	11.8 ± 6.9	0.07
HbA1c, %	8.5 ± 1.8	8.4 ± 1.8	8.6 ± 2.0	0.42
HbA1c, mmol/mol	69 ± 6	68 ± 6	70 ± 7	0.42
Retinopathy, %	357 (34.2)	341 (34.7)	16 (27.1)	0.24
Nephropathy, %	406 (57.3)	383 (57.5)	23 (53.5)	0.61
Peripheral arterial occlusive disease, %	119 (11.3)	110 (11.1)	9 (15.8)	0.27
Additional cardiovascular risk factors				
Family history of premature CAD, %	137 (13.0)	126 (12.7)	11 (19.0)	0.16
Hypertension, %	402 (76.3)	376 (76.3)	26 (76.5)	0.98
Lipid parameters				
Total cholesterol, mmol/L	4.5 ± 0.7	4.5 ± 0.7	4.7 ± 0.7	<0.05
HDL cholesterol, mmol/L	1.3 ± 0.5	1.3 ± 0.5	0.8 ± 0.1	<0.0001
Triglycerides, mmol/L	1.7 ± 1.0	1.6 ± 0.8	3.4 ± 1.1	<0.0001
LDL cholesterol, mmol/L	2.4 ± 0.6	2.4 ± 0.6	2.4 ± 0.6	0.27
Non HDL cholesterol, mmol/L	3.2 ± 0.7	3.1 ± 0.7	3.9 ± 0.7	<0.0001
Apoprotein A1, g/L	1.47 ± 0.74	1.49 ± 0.28	1.10 ± 0.12	<0.0001
Apoprotein B, g/L	0.89 ± 0.18	0.89 ± 0.18	1.10 ± 0.12	0.06
Fibrate treatment, %	96 (9.1)	87 (8.7)	9 (15.8)	0.09
Statin treatment, %	438 (41.4)	415 (41.5)	23 (40.4)	0.87
Smoking, %	245 (22.8)	226 (22.3)	19 (32.2)	0.08

Data are n (%) or mean ± SD

*CAD* coronary artery disease

**Table 2:** Characteristics of the patients who were included (LDL cholesterol <3.35 mmol/L) or not (LDL cholesterol ≥3.35 mmol/L) in the study

	Included patients n = 1080	Excluded patients n = 543	p
Clinical characteristics			
Age, years	61.6 ± 9.0	60.3 ± 9.2	0.09
Gender, male/female	614/466	291/252	0.21
Body mass index, kg/m^2^	30.2 ± 5.9	30.3 ± 6.4	0.786
Diabetes			
Diabetes duration, years	13.8 ± 8.5	13.7 ± 3.7	0.838
HbA1c, %	8.5 ± 1.8	8.9 ± 2.5	<0.001
Retinopathy, %	357 (34.2)	180 (34.0)	0.94
Nephropathy, %	406 (57.3)	181 (30.8)	<0.001
Peripheral neuropathy, %	233 (13.9)	139 (45.4)	0.71
Peripheral arterial occlusive disease, %	119 (11.3)	56 (10.4)	0.58
Family history of premature CAD, %	137 (13.0)	69 (13.4)	0.83
Additional cardiovascular risk factors			
Hypertension, %	402 (76.3)	235 (72.8)	0.25
Dyslipidemia, %	244 (65.3)	246 (72.1)	<0.01
Lipid parameters			
HDL cholesterol, mmol/L	1.3 ± 0.5	1.2 ± 0.4	0.001
Triglycerides, mmol/L	1.7 ± 1.0	1.9 ± 1.0	<0.001
LDL cholesterol, mmol/L	2.4 ± 0.6	4.1 ± 0.7	<0.001
Non-HDL cholesterol, mmol/L	3.2 ± 0.7	4.9 ± 0.9	<0.0001
Apoprotein A1, g/L	1.47 ± 0.29	1.50 ± 0.30	0.506
Apoprotein B, g/L	0.89 ± 0.18	1.31 ± 0.23	<0.0001
Fibrate treatment, %	96 (9.1)	61 (11.8)	0.085
Statin treatment, %	438 (41.4)	129 (25.0)	<0.001
Smoking, %	245 (22.8)	135 (25.1)	0.30

*CAD* coronary artery disease

Atherogenic dyslipidemia was diagnosed in 60 (5.6 %) patients with LDL <3.35 mmol/L (Table 1) and in 35 (6.0 %) patients with LDL <2.6 mmol/L (Table 3). In the study population, patients with atherogenic dyslipidemia had significantly higher mean total cholesterol and triglyceride levels, lower mean HDL cholesterol levels, higher non HDL cholesterol and lower apoprotein A1 levels, but did not differ significantly for the other characteristics from those without atherogenic dyslipidemia (Table 1). In the subgroup with LDL cholesterol <2.6 mmol/L, patients with atherogenic dyslipidemia were younger, had higher BMI, HbA1c and total cholesterol, a shorter diabetes duration and lower LDL cholesterol compared to patients without atherogenic dyslipidemia. They were also more likely treated by fibrate (Table 3).

**Table 3:** Characteristics of the patients with LDL cholesterol <2.6 mmol/L according to the presence of atherogenic dyslipidemia

	No atherogenic dysplipidemia n = 549	Atherogenic dysplipidemia n = 35	p
Clinical characteristics			
Age, years	61.6 ± 9.0	60.6 ± 7.4	0.001
Gender, male/female	334/215	24/11	0.36
Body mass index, kg/m^2^	30.1 ± 6.0	31.0 ± 5.0	0.001
Diabetes			
Diabetes duration, years	14.0 ± 8.4	12.0 ± 7.3	0.003
HbA1c, %	8.4 ± 1.9	9.0 ± 2.0	0.006
Retinopathy, %	180 (34.0)	10 (26.8)	0.51
Nephropathy, %	212 (60.1)	16 (69.6)	0.37
Peripheral neuropathy, %	111 (45.9)	10 (58.8)	0.30
Peripheral arterial occlusive disease, %	59 (11.2)	6 (18.2)	0.22
Family history of premature CAD, %	67 (12.5)	8 (23.5)	0.07
Additional cardiovascular risk factors			
Hypertension, %	207 (80.5)	13 (72.2)	0.39
Lipid parameters			
Total cholesterol, mmol/L	4.1 ± 0.6	4.4 ± 0.7	0.013
HDL cholesterol, mmol/L	1.4 ± 0.5	0.8 ± 0.1	0.073
Triglycerides, mmol/L	1.5 ± 0.9	3.7 ± 1.3	0.24
LDL cholesterol, mmol/L	2.0 ± 0.4	1.9 ± 0.4	0.003
Non-HDL cholesterol, mmol/L	2.7 ± 0.6	3.6 ± 0.7	<0.0001
Apoprotein A1, g/L	1.47 ± 0.29	1.13 ± 0.13	0.001
Apoprotein B, g/L	0.79 ± 0.16	0.91 ± 0.14	0.045
Fibrate treatment, %	41 (7.6)	6 (17.6)	<0.05
Statin treatment, %	279 (52.1)	15 (44.1)	0.37
Smoking, %	126 (23.1)	12 (34.2)	0.13

*CAD* coronary artery disease

### Stress tests results

In the total cohort, SMI was identified in 292 (27 %) patients. Among them, 218 subsequently underwent a coronary angiography, and CAD was confirmed in 91 patients (8.4 % of the total study population of 1080 patients and 41.7 % of the patients with SMI).

In the subgroup of 584 patients with LDL <2.6 mmol/L, SMI was diagnosed in 155 patients (26.5 %). Coronary angiography was performed in 112 subjects and CAD was confirmed in 45 patients.

### Parameters associated with silent myocardial ischemia

Table 4 shows that male gender, lower body mass index, retinopathy, POAD, higher mean blood pressure, smoking and atherogenic dyslipidemia including lower HDL cholesterol level were associated with SMI. In multivariate analyses taking into account all these parameters but HDL cholesterol, SMI was independently associated with atherogenic dyslipidemia (odds ratio 1.8 [95 % confidence interval 1.0–3.3], p < 0.05) (Table 5). In the subgroup of 584 patients with LDL <2.6 mmol/L, SMI was no longer associated with atherogenic dyslipidemia (prevalence in patients with vs without SMI: 7.7 vs 5.4 %, p = 0.323).

**Table 4:** Patients’ characteristics according to the presence or absence of silent myocardial ischemia or silent coronary artery disease in patients with LDL <3.35 mmol/L

	No SMI n = 788	SMI n = 292	Odds ratio^a^ [95CI]	p	No CAD n = 909	CAD n = 91	Odds ratio^b^ [95CI]	p
Clinical characteristics								
Age, years	61.6 ± 9.2	61.5 ± 8.6		0.90	61.4 ± 9.0	61.5 ± 8.7		0.93
Gender, male/female	405/383	209/83	2.4 [1.8–3.2]	<0.0001	486/423	73/18	3.5 [2.1–6.0]	<0.0001
Body mass index, kg/m^2^	30.6 ± 6.2	29.1 ± 5.1		<0.0001	30.5 ± 6.1	28.4 ± 4.4		<0.01
Diabetes								
Diabetes duration, years	13.8 ± 8.5	13.5 ± 8.6		0.56	13.8 ± 8.4	13.0 ± 8.5		0.39
HbA1c, %	8.4 ± 1.8	8.5 ± 2.0		0.43	8.5 ± 1.8	8.5 ± 2.1		0.95
HbA1c, mmol/mol	64 ± 6	65 ± 6		0.43	66 ± 6	65 ± 6		0.95
Retinopathy, %	243 (31.9)	114 (40.4)	1.4 [1.1–1.9]	0.01	290 (33.0)	41 (46.6)	1.8 [1.1–2.8]	<0.05
Nephropathy, %	294 (57.6)	112 (56.3)		0.74	340 (56.2)	36 (56.3)		0.99
Microalbuminuria >30 mg/day, %	205 (30.3)	88 (35.1)		0.17	242 (30.9)	35 (43.2)	1.7 [1.1–2.7]	<0.05
Peripheral arterial occlusive disease, %	60 (7.9)	59 (20.6)	3.0 [2.1–4.5]	<0.0001	75 (8.5)	23 (25.8)	3.8 [2.2–6.4]	<0.0001
Additional cardiovascular risk factors								
Hypertension, %	248 (74.3)	154 (79.8)		0.15	331 (75.2)	60 (81.1)		0.27
Mean blood pressure, mmHg	92 ± 11	94 ± 13		<0.01	92 ± 12	97 ± 13		<0.0001
Atherogenic dyslipidemia, %	36 (4.6)	24 (8.2)	1.9 [1.1–3.2]	<0.05	278 (61.6)	57 (75.0)	1.9 [1.1–3.2]	<0.05
Lipid parameters								
HDL cholesterol, mmol/L	1.3 ± 0.5	1.2 ± 0.4		<0.01	1.3 ± 0.5	1.1 ± 0.4		<0.0001
Triglycerides, mmol/L	1.7 ± 1.0	1.7 ± 0.9		0.48	1.7 ± 1.0	1.9 ± 1.1		<0.01
LDL cholesterol, mmol/L	2.4 ± 0.6	2.4 ± 0.6		0.97	2.4 ± 0.6	2.5 ± 0.6		0.34
Non HDL cholesterol, mmol/L	3.2 ± 0.7	3.2 ± 0.7		0.637	3.2 ± 0.7	3.3 ± 0.7		0.05
Apoprotein A1, g/L	1.47 ± 0.29	1.47 ± 0.25		0.958	1.48 ± 0.29	1.39 ± 0.22		0.195
Apoprotein B, g/L	0.89 ± 0.19	0.92 ± 0.18		0.205	0.89 ± 0.19	1.03 ± 0.15		0.002
Fibrate treatment, %	67 (8.7)	29 (10.2)		0.45	76 (8.5)	11 (12.2)		0.24
Statin treatment, %	316 (40.9)	122 (42.8)		0.57	365 (41.0)	39 (43.3)		0.66
Smoking, %	163 (20.8)	82 (28.3)	1.5 [1.1–2.0]	0.01	196 (21.7)	32 (35.2)	2.0 [1.2–3.1]	<0.01

Data are nη or mean ± SD

*CAD* coronary artery disease, *SMI* silent myocardial ischemia, *95CI* confidence interval at 95 %

^a^Odds ratio comparing patients with SMI to those without

^b^Odds ratio comparing the patients with CAD to those without

**Table 5:** Parameters explaining silent myocardial ischemia and coronary artery disease in multivariate analyses

	Odds ratio	95 % confidence interval	p
Silent myocardial ischemia in patients with LDL <3.35 mmol/L: H Lemershow: 7.35, p = 0.499			
Male gender	2.1	1.5–2.9	<0.001
Body mass index	0.97	0.94–0.997	<0.05
Retinopathy	1.4	1.1–1.9	<0.05
Peripheral occlusive arterial disease	2.5	1.6–3.8	<0.001
Mean blood pressure	1.01	1.00–1.03	0.01
Atherogenic dyslipidemia	1.8	1.0–3.3	0.05
Smoking			NS
Silent coronary artery disease in patients with LDL <3.35 mmol/L: H Lemershow: 5.30, p = 0.725			
Male gender	3.0	1.6–5.6	<0.0001
Body mass index	0.94	0.90–0.995	<0.05
Retinopathy	1.7	1.0–2.9	<0.05
Microalbuminuria			NS
Peripheral occlusive arterial disease	4.0	2.1–7.4	<0.0001
Mean blood pressure	1.03	1.01–1.05	0.001
Atherogenic dyslipidemia	4.0	1.7–9.2	0.001
Smoking			NS
Silent coronary artery disease in patients with LDL <2.6 mmol/L: H Lemershow: 12.85, p = 0.117			
Male gender	3.0	1.1–8.2	<0.05
Body mass index			0.07
Retinopathy			0.09
Peripheral occlusive arterial disease	3.7	1.7–8.1	0.001
Mean blood pressure	1.03	1.00–1.06	<0.05
Atherogenic dyslipidemia	2.96	0.97–9.03	0.057
Smoking	2.2	1.1–4.4	<0.05

### Parameters associated with silent coronary artery disease

Male gender, lower body mass index, retinopathy, microalbuminuria, POAD, higher mean blood pressure, smoking and atherogenic dyslipidemia including lower HDL cholesterol and higher triglyceride levels, higher non-HDL cholesterol and apoprotein B levels were associated with silent CAD (Table 4). In multivariate analyses taking into account all these parameters but HDL cholesterol, triglycerides levels, non-HDL cholesterol and apoprotein B; silent CAD was independently associated with atherogenic dyslipidemia (OR 4.0 [1.7–9.2], p < 0.001) (Table 5).

In patients with LDL <2.6 mmol/L, CAD was associated with male gender (OR 4.7 [1.9–11.2], p < 0.0001), lower body mass index (p < 0.01), POAD (OR 5.3 [2.6–10.7]), higher mean blood pressure (p < 0.05), atherogenic dyslipidemia (prevalence in patients with vs without CAD 15.6 vs 4.8 %, OR 3.6 [1.5–9.0], p = 0.01) including HDL cholesterol (p < 0.01), and smoking (OR 2.7 [1.5–5.1], p < 0.001); with a trend for retinopathy (p = 0.09) (Table 6). Multivariate analysis taking into account these parameters but HDL-cholesterol showed that atherogenic dyslipidemia was not significantly associated (p = 0.057) with silent CAD in patients with LDL <2.6 mmol/L (Table 5).

**Table 6:** Characteristics of the patients with LDL cholesterol <2.6 mmol/L according to the presence of asymptomatic coronary artery disease

	No CAD n = 496	CAD n = 45	Odds ratio [95CI]	p
Clinical characteristics				
Age, years	61.3 ± 8.9	61.1 ± 8.4		NS
Gender, male/female	289/207	39/6	4.7 [1.9–11.2]	<0.0001
Body mass index, kg/m^2^	30.5 ± 6.1	27.6 ± 3.7		<0.01
Diabetes				
Diabetes duration, years	14.0 ± 8.2	12.6 ± 7.5		NS
HbA1c, %	8.4 ± 1.9	8.5 ± 1.9		NS
Retinopathy, %	157 (32.8)	20 (45.5)		0.09
Nephropathy, %	193 (59.9)	17 (53.1)		NS
Microalbuminuria >30 mg/day, %	151 (35.4)	16 (39.0)		NS
Creatinine clearance, ml/min	86.6 ± 28.6	84.8 ± 26.2		NS
Urinary albumin excretion rate, mg/day	136.3 ± 506.7	139.1 ± 228.3		NS
Proteinuria, g/day, %	0.4 ± 1.0	0.3 ± 0.3		NS
Peripheral neuropathy, %	98 (44.5)	16 (53.3)		NS
Peripheral arterial occlusive disease, %	40 (8.4)	14 (32.6)	5.3 [2.6–10.7]	<0.0001
Additional cardiovascular risk factors				
Hypertension, %	180 (79.3)	33 (82.5)		NS
Mean blood pressure, mmHg	92.0 ± 11.1	96.3 ± 11.4		<0.05
Atherogenic dyslipidemia, %	24(4.8)	7(15.6)	3.6 [1.5–9.0]	0.01
Lipid parameters				
HDL cholesterol, mmol/L	1.3 ± 0.5	1.1 ± 0.3		<0.01
Triglycerides, mmol/L	1.7 ± 1.0	1.9 ± 1.3		NS
LDL cholesterol, mmol/L	2.0 ± 0.4	2.0 ± 0.5		NS
Non-HDL cholesterol, mmol/L	2.7 ± 0.6	2.9 ± 0.7		0.224
Apoprotein A1, g/L	1.46 ± 0.30	1.42 ± 0.24		0.706
Apoprotein B, g/L	0.80 ± 0.16	0.92 ± 0.14		0.034
Fibrate treatment, %	38 (7.9)	5 (11.1)		NS
Statin treatment, %	250 (51.7)	24 (53.3)		NS
Smoking, %	111 (22.6)	20 (44.4)	2.7 [1.5–5.1]	<0.01

*CAD* coronary artery disease

**Fig. 1:**
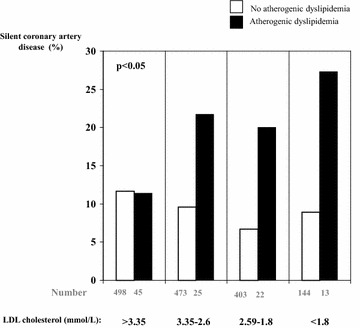
Prevalence of silent coronary artery disease according to presence of atherogenic dyslipidemia and LDL cholesterol levels

Finally, the association between atherogenic dyslipidemia and silent CAD was only shown for patients with LDL cholesterol <3.35 mmol/L (Fig. 1).

## Discussion

The results of this cohort study show that atherogenic dyslipidemia is significantly and independently associated with asymptomatic CAD in patients with type 2 diabetes, especially those with LDL cholesterol below 3.35 mmol/L. Figure 1 again illustrates the potential role of atherogenic dyslipidemia in the residual cardiovascular risk. These findings are strengthened by the size of the cohort and consistency with other reports for the prevalence of SMI in this setting [3, 7, 27].

### Prevalence of atherogenic dyslipidemia

The prevalence of atherogenic dyslipidemia was lower than anticipated from the ACCORD Lipid study population (17 %), which was also characterized by long-standing diabetes and LDL cholesterol levels in the target range [20]. This was not unexpected. First the ACCORD Lipid study defined the subgroup with atherogenic dyslipidemia using tertile analysis [20], with thresholds that were different from the tertiles in our cohort: 0.88 mmol/L in ACCORD and 1.07 mmol/L in our series for HDL cholesterol, and 2.26 and 1.86 mmol/L respectively for triglycerides. Second, high triglycerides and low HDL cholesterol are biologically associated [23]. An analysis of the ACCORD Lipid study suggested a more conservative estimate of ~10 % [28]. In a population of subjects with diabetes from Europe and Canada treated by statins, the rate of patients with both LDL cholesterol below target value (<2.6 mmol/L) and low HDL cholesterol plus elevated triglycerides was 6 % [29]. Thus, the current study provides a more accurate estimation of the true prevalence of this dyslipidemic profile in a real-life clinical setting, in which a substantial proportion of high-risk patients with type 2 diabetes was treated with statins.

### Association between atherogenic dyslipidemia and SMI

We show here for the first time an association between atherogenic dyslipidemia and silent CAD. This association was found in individuals with type 2 diabetes and LDL cholesterol lower than 3.35 mmol/L with a borderline significance for those with LDL cholesterol lower than 2.6 mmol/L. The role of atherogenic dyslipidemia on incident cardiovascular events is well-known [30–32] but to our knowledge, few studies have previously reported an association between atherogenic dyslipidemia and prevalent cardiovascular diseases, such an association between a high triglyceride-to-HDL cholesterol ratio and left ventricular geometry in children [33] and CAD in type 2 diabetes [16]. Atherogenic dyslipidemia has also been reported to be usual in Indian patients with proven CAD [34]. Some studies have reported an association between high levels of triglycerides and SMI [17, 18] and CAD [18]; and between low levels of HDL-cholesterol and CAD [19]. However, these observations were not analyzed according to the combination of lipid parameters, statin and/or fibrate treatments, or LDL cholesterol levels. This appears to be important as our data did not show any association between atherogenic dyslipidemia and SMI in patients with LDL cholesterol above 3.35 mmol/L (Fig. 1). Atherogenic dyslipidemia might therefore contribute to residual cardiovascular risk in type 2 diabetes. Altogether, considering atherogenic dyslipidemia as a marker or a risk factor for SMI and silent CAD should be limited to the patients with controlled LDL cholesterol. However, this might have therapeutic implications.

### Therapeutic implications

Whether the identification of patients with diabetes and SMI may reduce their cardiovascular risk has been debated [6, 35–37]. However, treating atherogenic dyslipidemia was never considered in these studies. Based on our findings, we propose that targeting atherogenic dyslipidemia might help to reduce the residual risk of asymptomatic CAD. In support, an analysis of the Fenofibrate Intervention and Event Lowering in Diabetes (FIELD) study showed that treatment with fenofibrate (which lowers triglycerides and raises HDL cholesterol) led to a 78 % reduction in subsequent cardiovascular events in patients with silent myocardial infarction (p = 0.0003) [38]. Additionally, in the Diabetes Atherosclerosis Intervention Study (DAIS), fenofibrate reduced progression of CAD in patients with type 2 diabetes, about 50 % of them having clinical disease [25]. A treatment with fenofibrate versus placebo added to statin therapy in patients with type 2 diabetes was associated with a 30 % reduction of cardiovascular events in subjects with atherogenic dyslipidemia as defined as in our cohort [20]. Further evidence supporting the clinical benefits of fibrate-based therapy in patients with atherogenic dyslipidemia was provided by a meta-analysis of five placebo-controlled studies, which showed that fibrate use was associated with a 35 % reduction in the risk of coronary events [39]. Long-term mortality was also shown to be reduced in patients with CAD who were initially allocated to bezafibrate versus placebo and this effect was more prominent in those with triglyceride levels ≥200 mg/dL [26]. In the patients with atherogenic dyslipidemia, achieving target non-HDL cholesterol levels should be a key focus of cardio-vascular risk management [40]. Non-HDL cholesterol is well defined as a secondary target in the treatment of dyslipidemias in the European Atherosclerosis Society/European Society of Cardiology (EAS/ESC) guidelines, which set a specific target 0.8 mmol/L higher than the corresponding LDL cholesterol target value [41]. Such was the case in our subpopulation with silent CAD in which subjects had non-HDL cholesterol level 0.88 mmol/L higher than LDL cholesterol (Table 4). Non-HDL cholesterol, and similarly apoprotein B, were associated with an increased risk of silent CAD. Therefore, non-HDL cholesterol should be an additional target in this very high-risk population.

### Limitations

We acknowledge a number of limitations to this study. These include the observational design of the study, which cannot show a causal relationship but only an association between atherogenic dyslipidemia and SMI, the inclusion of inpatients and a low prevalence of atherogenic dyslipidemia. Mean LDL cholesterol level in our series was 2.4 mmol/L but the absolute number of patients with atherogenic dyslipidemia who had LDL cholesterol level strictly below the target value of 2.6 mmol/L was low. Furthermore, only 41 % of the patients were treated with statin, which is nowadays routinely prescribed in patients with type 2 diabetes. However, we report one of the largest ever-published series of asymptomatic patients with type 2 diabetes, and known CAD status, although some patients with SMI did not undergo a coronary angiography. Despite these limits, the results of the study suggest that atherogenic dyslipidemia might be a contributing factor to the residual burden of cardiovascular disease in patients with type 2 diabetes, in line with recent expert consensus [40].

In conclusion, the present study demonstrates that atherogenic dyslipidemia is associated with increased risk of both SMI and silent CAD in high-risk patients with type 2 diabetes, especially in those with LDL cholesterol lower than 3.35 mmol/L. A patient screening taking this factor into account might help with an earlier identification of asymptomatic CAD. Since a significant part of the residual risk is related both to atherogenic dyslipidemia and silent coronary disease, targeting triglycerides and HDL cholesterol might be considered in patients with silent CAD and such lipid disorders. However, our findings need to be confirmed by prospective studies conducted in larger series of patients with atherogenic dyslipidemia and with LDL cholesterol in the target range.

## Abbreviations

CAD: coronary artery disease
SMI: silent myocardial ischemia
POAD: peripheral occlusive arterial disease
